# Natural Nanoparticles in Gegen–Qinlian Decoction Promote the Colonic Absorption of Active Constituents in Mice with Dextran Sulfate Sodium-Induced Ulcerative Colitis

**DOI:** 10.3390/ph18111718

**Published:** 2025-11-12

**Authors:** Sheng Mu, Zhang-Jin Zheng, Jing-Ze Lu, Ling-Yun Pan, Bing-Liang Ma

**Affiliations:** 1 Department of Pharmacology, School of Pharmacy, Shanghai University of Traditional Chinese Medicine, Shanghai 201203, China; musheng0925@163.com (S.M.); zhangjinzheng2025@163.com (Z.-J.Z.); jingze_lu97@163.com (J.-Z.L.); 2 Experiment Center for Science and Technology, Shanghai University of Traditional Chinese Medicine, Shanghai 201203, China

**Keywords:** natural nanoparticles, Gegen–Qinlian decoction, ulcerative colitis, active constituents, pharmacokinetics, drug delivery system

## Abstract

**Background/Objectives**: The aim of this study was to reveal the influence of the natural nanoparticles (Nnps) isolated from Gegen–Qinlian Decoction (GQD), i.e., GQD-Nnps, on the intestinal absorption and pharmacokinetic properties of several representative active GQD constituents. **Methods**: The morphology of GQD-Nnps was examined using scanning electron microscopy (SEM). Protein and polysaccharide contents were measured using the bicinchoninic acid (BCA) assay and phenol–sulfuric acid method, respectively. Major GQD constituents were quantified by liquid chromatography–tandem mass spectrometry (LC-MS/MS). Formation mechanisms were explored using dynamic light scattering (DLS), Fourier transform infrared spectroscopy (FTIR), and high-resolution mass spectrometry (HRMS). Pharmacokinetic studies were conducted in mice with dextran sulfate sodium (DSS)-induced UC. **Results**: GQD-Nnps were spherical, with a size of 110.9 ± 8.1 nm and a zeta potential of −13.7 ± 1.5 mV. GQD-Nnps were primarily composed of proteins and polysaccharides. FTIR analysis revealed significant hydrogen bonding interactions between the small molecular and macromolecular constituents of GQD. HRMS analyses indicated complex formation among small molecules, particularly berberine, baicalin, and glycyrrhizic acid. DLS demonstrated good stability of GQD-Nnps in artificial gastric and intestinal fluids. Pharmacokinetic studies showed that, except for puerarin, blood and liver exposure levels of several constituents in the GQD-Nnps group were significantly higher than those in the GQD extract group, suggesting enhanced colonic absorption and hepatic distribution. **Conclusions**: GQD-Nnps create an oral drug delivery system through complex interactions, significantly enhancing the colonic absorption and hepatic distribution of several active GQD constituents.

## 1. Introduction

Ulcerative colitis (UC) is an inflammatory bowel disease (IBD) characterized by persistent superficial mucosal inflammation that originates in the rectum and extends proximal to the colon [1]. Up to 50% of IBD patients experience at least one extraintestinal manifestation (EIM), such as hepatobiliary diseases [2], and are at an increased risk of developing colon cancer [3]. In 2021, there were 375,140 new cases and 3.83 million total cases of IBDs [4]. 5-aminosalicylates (5-ASAs), corticosteroids, immunomodulators and biological agents such as anti-tumor necrosis factor agents are commonly used to treat adult UC patients [5].

After oral administration, most of the drug is absorbed in the small intestine, making it challenging to reach the distal colon. To enhance anti-UC efficacy in UC primarily affecting the rectum and sigmoid colon, 5-ASAs and other drugs are often administered rectally [5]. However, this route of administration is poorly accepted by some patients [5]. In addition, oral administration is more appropriate for UC that affects the higher portions of the colon [6]. Nano-drug delivery systems are used for colon-targeted drug delivery to enhance the efficacy of anti-UC drugs [6]. For instance, natural polymers, such as polysaccharides, are employed to create colon-targeted oral nanoparticles due to their ease of use, low cost, and high bio-adhesion and biodegradability [7]. The stability of nanoparticles during transport following oral administration is crucial for the effective delivery of the drug to the colon [6,7].

The Chinese Guidelines for the Diagnosis and Treatment of Ulcerative Colitis (2023) recommend traditional Chinese medicines (TCMs) for mild-to-moderate left-sided active UC due to their multi-component and multi-target characteristics and low risk of adverse reactions [8]. Gegen–Qinlian decoction (GQD) is a classic TCM prescription made up of Puerariae Lobatae Radix, Scutellariae Radix, Coptidis Rhizoma, and Glycyrrhizae Radix et Rhizoma Praeparata cum Melle in an 8:3:3:2 mass ratio [9]. Clinically, GQD alone can alleviate UC symptoms and prevent recurrence [10]. It can also synergize the efficacy of modern medications [10]. In model mice, oral administration of GQD dramatically improved UC by reducing inflammation and oxidative stress, restoring colonic mucosal homeostasis, and modulating gut microbiota [11,12,13,14,15,16,17]. Furthermore, GQD has a number of other pharmacological effects, including liver protection and anti-lung injury [9], implying that it may relieve the EIMs of UC.

After oral administration of GQD water extract, six constituents, including puerarin, berberine, baicalin, baicalein (the main metabolite of baicalin [18]), glycyrrhizic acid, and glycyrrhetinic acid (the main metabolite of glycyrrhizic acid [19]), had high exposure levels in the colon and liver of mice [20]. It has been reported that each of these constituents has potential anti-UC effect [21,22,23,24]. Furthermore, a mixture of GQD small molecules composed primarily of these constituents relieved UC via anti-inflammatory and antioxidant actions [25]. These findings suggest that the constituents may serve as the effective material basis for the anti-UC effects of GQD. However, the constituents, including puerarin [26], baicalin [27], berberine [28], and glycyrrhizic acid [29], have poor permeability and metabolic stability. Consequently, the development of colon-targeted oral formulations of the active constituents is expected to enhance their pharmacokinetic properties and anti-UC effects.

TCM extracts commonly contain natural nanoparticles (Nnps) produced by self-assembly of constituents [30]. For example, Nnps isolated from the extracts of *Coptis chinensis* and *Ganoderma lucidum* increase the oral absorption and pharmacokinetic properties of berberine and docetaxel, respectively [31,32,33]. It was reported that GQD contains micro- and nano-sized aggregates, which have anti-diabetic effects and can improve the intestinal absorption of baicalin [34]. We assumed that Nnps in GQD (GQD-Nnps) may affect the intestinal absorption and tissue distribution of the active GQD constituents.

In this study, Nnps were isolated from GQD under optimized conditions, and their composition and formation mechanism were explored. Next, the pharmacokinetics of GQD-Nnps were studied in dextran sulfate sodium (DSS)-induced UC mice and compared with GQD water extract and a mixture of four GQD main constituents. This study aimed to reveal the characteristics and potential advantages of GQD-Nnps in the treatment of UC and would provide an experimental basis for the further development and utilization of GQD and its constituents.

## 2. Results

### 2.1. Yield and Quality Control of GQD Extract

A total of 992 g of herbal pieces were subjected to decoction, resulting in approximately 279 g of GQD extract powder, which corresponds to a yield of 28.1%.

Within the GQD extract powder, the contents of berberine, puerarin, baicalin, and glycyrrhizic acid were determined to be 17.75 ± 1.39, 40.50 ± 3.54, 45.43 ± 2.83, and 4.52 ± 0.60 mg/g, respectively.

### 2.2. Optimized Isolation Protocol for GQD-Nnps

The results of the orthogonal experiments shown in Table 1 indicated that the initial concentration of GQD extract, dialysis time, and stirring speed had weak effects on the size and zeta potential of GQD-Nnps, but significantly impacted the contents and encapsulation efficiency of the four representative constituents in GQD-Nnps.

**Figure 1:**
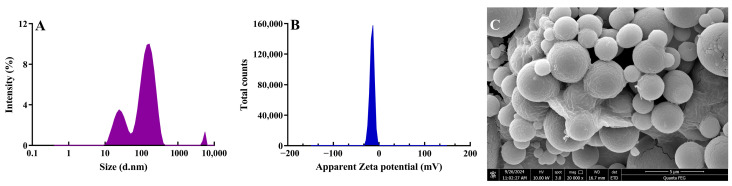
Representative results of dynamic light scattering analysis (**A**,**B**) and scanning electron microscopy observations (SEM) of GQD-Nnps. (**A**), size; (**B**), zeta potential; (**C**), SEM observation.

According to the results, the initial concentration of GQD extract was determined to be 200 mg/mL, the dialysis time was 24 h, and the stirring speed was 200 rpm. Under the optimized condition, the prepared GQD-Nnps had a size of 110.9 ± 8.1 nm, a polydispersity index (PDI) of 0.45 ± 0.08, and a zeta potential of −13.7 ± 1.5 mV. Figure 1 shows the representative results of DLS analysis for GQD-Nnps.

### 2.3. Yield of GQD-Nnps

About 22.8 g of GQD-Nnps powder was obtained from 144 g of GQD extract powder based on the optimized isolation protocol, with a yield of 15.8%.

### 2.4. Contents of Protein and Polysaccharide in GQD-Nnps

According to the BCA and phenal–sulfuric acid assays, the protein content of GQD-Nnps was about 95.6 ± 2.9%, while the polysaccharide content was about 50.4 ± 1.1%, indicating that GQD-Nnps were mainly composed of proteins.

### 2.5. Morphology and Size of Lyophilized GQD-Nnps

SEM observations showed that lyophilized GQD-Nnps were spherical and non-uniform in size (Figure 1C).

### 2.6. In Vitro Stability of Lyophilized GQD-Nnps

**Figure 2:**
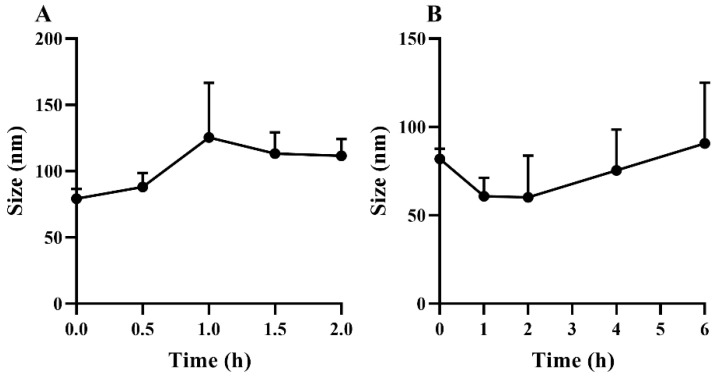
Size of GQD-Nnps after incubation in artificial gastric fluid (**A**) and artificial intestinal fluid (**B**), respectively (mean ± SD, n = 3). The groups at time point “0” were the control groups.

The size of GQD-Nnps remained unchanged within 0.5 h but slightly increased after longer period of incubation in the artificial gastric fluid (Figure 2A). However, after incubation for 2 h, the size of GQD-Nnps was kept below 150 nm, indicating its good stability in the stomach post-oral administration. Similarly, after incubation for 6 h in the artificial intestinal fluid, the size of GQD-Nnps remained around 100 nm (Figure 2B), indicating their good stability in the intestine post-oral administration.

### 2.7. FTIR Analysis

**Figure 3:**
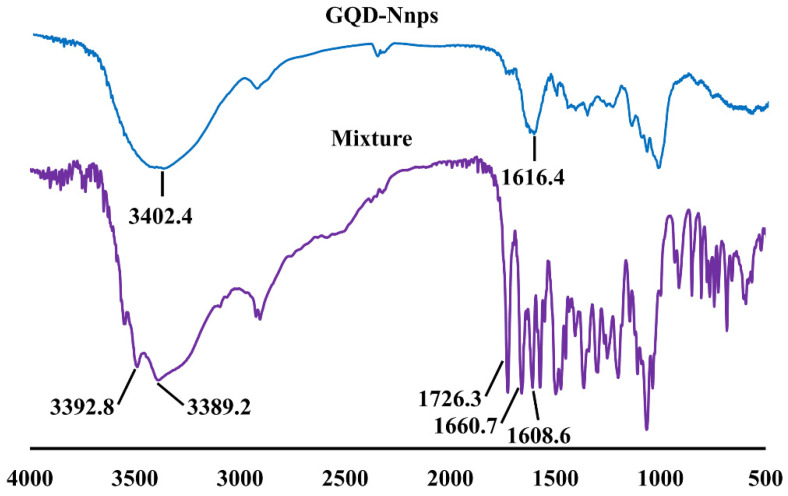
Fourier transform infrared spectroscopy of the GQD mixture (composed of berberine, baicalin, puerarin, and glycyrrhizic acid) and GQD-Nnps.

The FTIR spectra of the GQD mixture (containing berberine, baicalin, puerarin, and glycyrrhizic acid) and GQD-Nnps are presented in Figure 3. In the FTIR spectrum of GQD-Nnps, the peak at 3402.4 cm^−1^ (reflecting O-H and N-H stretching) exhibited a significant shift compared to those in the spectra of the GQD mixture (3392.8 and 3389.2 cm^−1^). In addition, the characteristic peaks reflecting C=O stretching in the GQD mixture (1726.3, 1660.7, and 1608.6 cm^−1^) all disappeared in the spectrum of Nnps-DTX, which had a peak of 1616.4. Therefore, it can be concluded that hydrogen bond interactions occurred in GQD-Nnps.

### 2.8. HRMS Analysis

**Figure 4:**
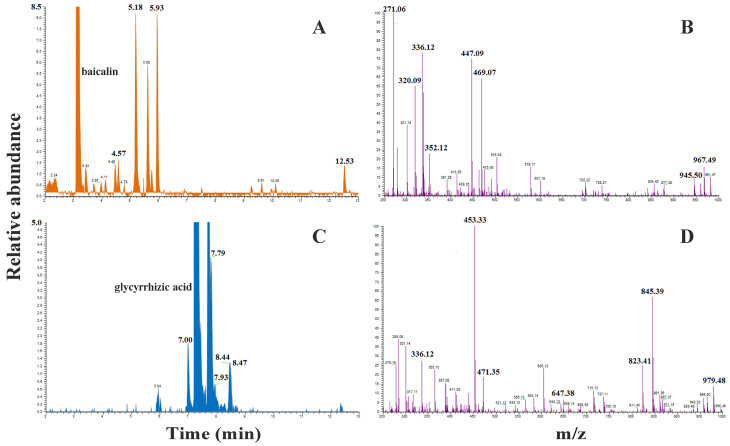
High-resolution mass spectrometry of the complexes of berberine–baicalin and berberine–glycyrrhizic acid in GQD-Nnps.

HRMS detection confirmed the formation of stable complexes between berberine and both baicalin and glycyrrhizic acid in GQD-Nnps (Figure 4). For example, the berberine–baicalin complex was identified by a peak in the total ion chromatogram (TIC) of baicalin at retention time 5.18 min, with corresponding fragment ions at *m*/*z* 336.12 (berberine) and 447.09 (baicalin). Similarly, the berberine–glycyrrhizic acid complex was evidenced by a peak in the TIC of glycyrrhizic acid at retention time 7.70 min, with fragment ions at *m*/*z* 336.12 (berberine) and 823.41 (glycyrrhizic acid).

### 2.9. Pharmacokinetics of the Constituents of Oral GQD in Mice

#### 2.9.1. Induction of UC in Mice

On the seventh day of modeling, the average body weight of the mice decreased by approximately 12% (20.6 ± 1.3 g vs. 18.2 ± 0.9 g, *p* < 0.01), indicating successful induction of UC in the mice.

#### 2.9.2. Pharmacokinetics of the Constituents in the Systemic Circulation

**Figure 5:**
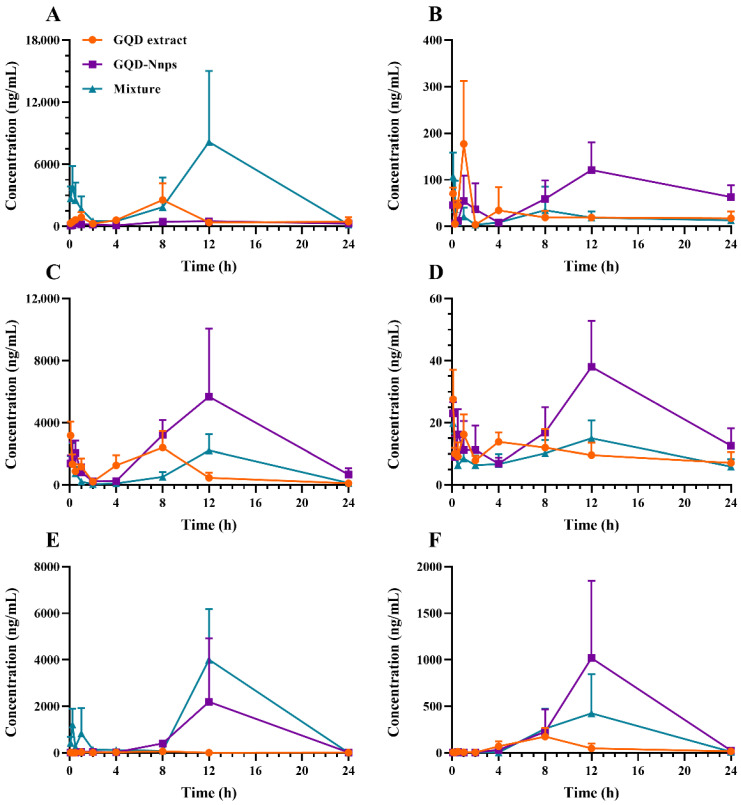
Concentration–time curves of six GQD constituents in the systemic circulation of the mice after oral administration of the GQD extract, the GQD mixture (composed of berberine, baicalin, puerarin, and glycyrrhizic acid), or GQD-Nnps, respectively (mean ± SD, n = 5). (**A**–**F**), puerarin, berberine, baicalin, baicalein, glycyrrhizic acid, and glycyrrhetinic acid, respectively.

The complete concentration–time (C-T) curves of four representative parent GQD constituents and two metabolites within the systemic circulation are depicted in Figure 5, with the corresponding pharmacokinetic parameters detailed in Table 2.

A double-peak pattern was noted for each constituent across all groups, characterized by a second peak at 8 or 12 h post-administration, suggesting the constituents’ colonic absorption. Notably, this second peak was significantly higher than the first, indicating a predominant role of the colon in intestinal absorption of GQD constituents during UC. In addition, the time to reach the second absorption peak was delayed in the GQD-Nnps and the mixture groups compared to the GQD extract group. In terms of exposure level, the GQD extract group exhibited the lowest concentrations for all constituents. In contrast, the mixture group showed the highest exposure level for puerarin and glycyrrhizic acid, whereas the GQD-Nnps group demonstrated the highest exposure level for berberine, baicalin, baicalein, and glycyrrhetinic acid.

#### 2.9.3. Pharmacokinetics of the Constituents in the Livers

**Figure 6:**
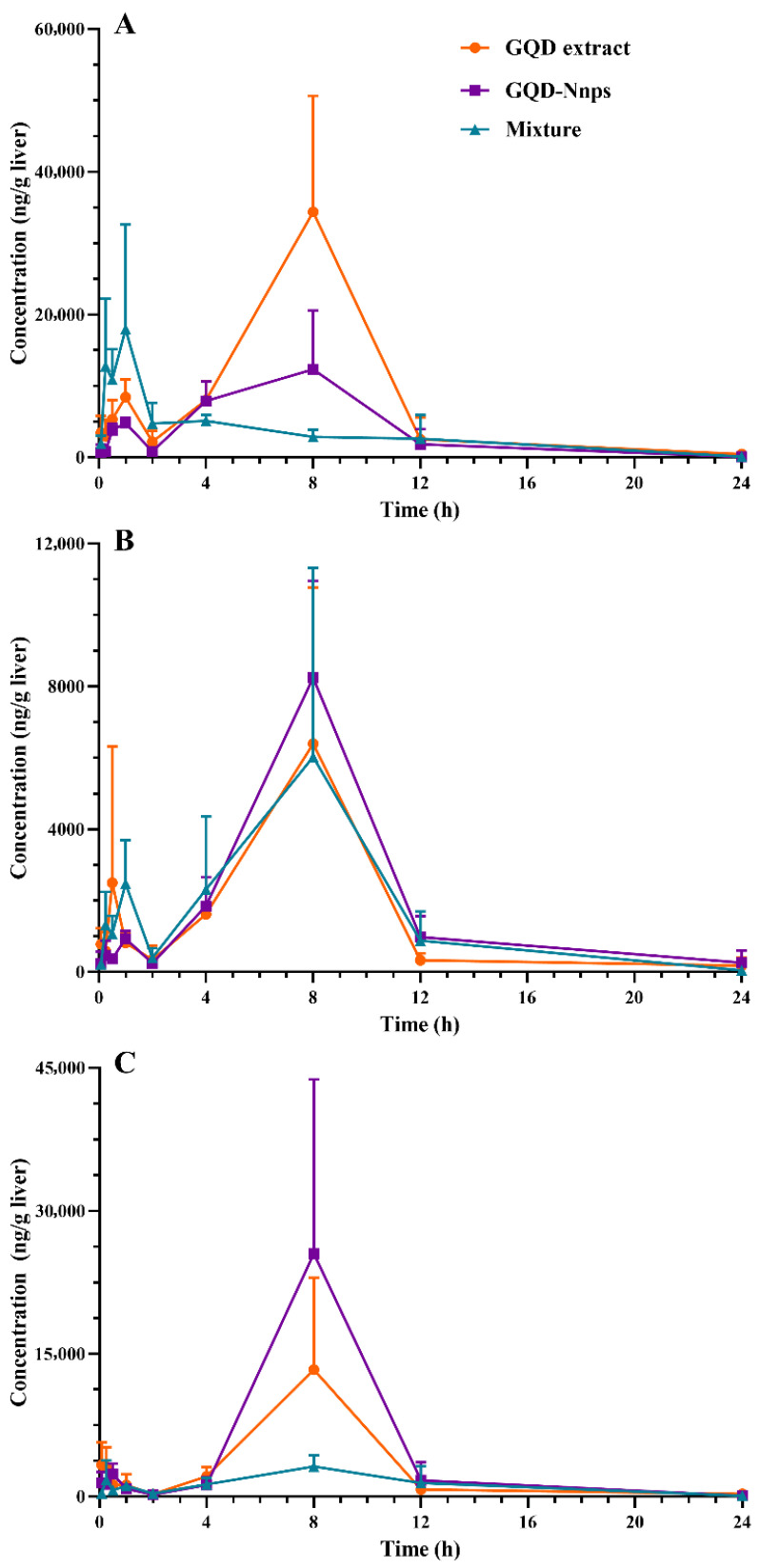
Concentration–time curves of three GQD constituents in the livers of the mice after oral administration of the GQD extract, the GQD mixture (composed of berberine, baicalin, puerarin, and glycyrrhizic acid), or GQD-Nnps, respectively (mean ± SD, n = 5). (**A**–**C**), puerarin, berberine, and baicalein, respectively.

Figure 6 shows that only puerarin, berberine, and baicalein could produce complete C-T curves in the liver. Table 2 shows the pharmacokinetic parameters in detail. Similar to the systemic circulation, the C-T curves of these constituents showed two peaks, and the second peak (also the highest peak) appeared almost at 8 h after administration. The GQD extract group had the highest exposure level to puerarin, whereas the GQD-Nnps group had the highest exposure to baicalein.

## 3. Discussion

In addition to small molecular compounds, there are also high contents of proteins and polysaccharides in TCM extracts. These macromolecules can self-assemble to form Nnps, which act as carriers to adsorb or encapsulate small molecular compounds, thereby altering their pharmacological and pharmacokinetic properties [30]. Nnps containing both macromolecular and small molecular constituents can be considered as the functional units through which TCM exerts its effects [30]. However, it is challenging to isolate Nnps quickly and efficiently from TCM extracts. A straightforward method has been developed for the isolation of Nnps through centrifugation, filtration, and dialysis [31,32], which significantly enhances efficiency compared to size-exclusion high-performance liquid chromatography [35]. However, the isolation conditions must be refined to ensure that the obtained Nnps have the ideal size and zeta potential and the small molecular active constituents within the Nnps maintain an appropriate content and encapsulation efficiency. In this study, orthogonal experimental design was adopted, and it was found that different initial extract concentration, dialysis time, and stirring speed had little effect on the size and zeta potential of Nnps, but had significant effects on the content and encapsulation efficiency of four GQD constituents within Nnps.

In general, lyophilization may increase the particle size of nanoparticles, necessitating the use of a lyophilizer. However, in this study, the prepared GQD-Nnps exhibited favorable particle size and Zeta potential. Thus, no lyoprotectant was added to avoid its potential adverse effects.

The results of BCA and phenol sulfate analysis showed that GQD-Nnps are primarily composed of proteins and polysaccharides. The combined content of these constituents exceeds 100%, a phenomenon also observed in Nnps from *Coptis chinensis* [31,33] and *Ganoderma lucidum* [32], likely due to the insufficient specificity of the detection method. It has been documented that carbohydrates may interfere with the BCA assay [36]. SEM observations reveal that GQD-Nnps exhibit a spherical shape and are heterogeneous in size, which is a common characteristic of Nnps [31,32,33]. It should be noted that SEM was used to observe the morphology in lyophilized powder, where nanoparticle aggregation was unavoidable, leading to increased particle size. In contrast, DLS analyzed the particle size after dispersion in solution, which represents the true size of the nanoparticles. In addition, Figure 1 and Figure 2 also show differences in particle size, which we speculate may be due to different dispersion media: the former was dispersed in pure water, while the latter was dispersed in simulated gastric fluid and intestinal fluid. We believe that the particle size of the nanoparticles during intestinal transport is less than the 200 nm cutoff value. This size advantage facilitates their intestinal absorption, as particle size is a critical factor for the absorption of orally administered nanoparticles [6]. The zeta potential of GQD-Nnps suggests the potential for aggregation, indicating that it is preferable to administer them promptly after aqueous dispersion. Surprisingly, although mainly composed of protein, GQD-Nnps were very stable in artificial gastric fluid and artificial intestinal fluid containing digestive enzymes. It has been reported that polysaccharides are capable of forming complexes with proteins through electrostatic and hydrophobic interactions [37]. In addition, polysaccharide modification helps to increase the stability of protein nanoparticles [38]. However, whether the proteins and polysaccharides in GQD-Nnps form complexes remains to be confirmed.

FTIR analysis revealed significant hydrogen bonding interactions between the small molecular and macromolecular constituents of GQD-Nnps. Additionally, HRMS analyses indicated that the small molecules, particularly berberine, baicalin, and glycyrrhizic acid, formed complexes. These findings suggest that various intermolecular interactions promote the formation of GQD-Nnps. While previous studies have reported the complexation of berberine with baicalin and glycyrrhizic acid, leading to the self-assembly of carrier-free nanoparticles [39], this study clearly demonstrates that complex formation plays a role in the development of Nnps in TCM extract.

Pharmacokinetic studies have shown that, except for puerarin, the blood and liver exposure levels of several constituents in the GQD-Nnps group were significantly higher than those in the GQD extract group, indicating that GQD-Nnps can promote intestinal absorption and tissue distribution of the constituents. Puerarin in GQD-Nnps does not have significant interactions with other constituents and cannot be effectively encapsulated or adsorbed by Nnps, resulting in a low loading and poor encapsulation efficiency. In healthy mice, the C-T profiles of GQD constituents in blood and tissues exhibited a single peak at 4 h post-oral administration [20], contrasting with this study’s findings, which revealed a biphasic peak pattern with a peak time of 8 or 12 h. Following oral administration, drugs typically take three to four hours to pass through the small intestine and six hours to reach the colon [40,41]. Therefore, a peak at 8 or 12 h suggests that the colon is where the drug is absorbed. GQD-Nnp is stable during gastrointestinal transit and can be delivered to the colon and absorbed, thereby generating a secondary peak on the C-T curves. Furthermore, the pharmacokinetic data suggest that absorption occurs primarily in the colon, with limited uptake in the small intestine. We hypothesize that because GQD-Nnps exhibit relative stability during intestinal transit and the small intestinal structure remains intact in the UC model, only compounds released from GQD-Nnps are absorbed in the small intestine. In the colon, however, the disrupted intestinal barrier allows GQD-Nnps to be directly absorbed in the form of nanoparticles, which significantly facilitates absorption [42]. Nevertheless, the exact mechanism remains to be elucidated. The increased in vivo exposure levels of active constituents are beneficial for their therapeutic effects on the EIMs of UC, and the GQD-Nnps delivered to the colon may regulate the intestinal microbiota and protect epithelial cells from damage. The blood drug concentrations of puerarin and glycyrrhizic acid in the mixture group were higher than those in the extract group, indicating that the administration of small-molecule mixtures is also feasible for certain constituents. Compared to the GQD extract, GQD-Nnps may inhibit the intestinal metabolism and efflux of constituents like berberine, while compared to the constituent mixture, GQD-Nnps may reduce the size of the constituents, which usually exist in the form of larger crystals [30,31]. These combined effects likely contribute to the improved pharmacokinetic profile observed with GQD-Nnps.

This study demonstrated GQD-Nnps’ unique composition, formation mechanism, and pharmacokinetic properties. Nonetheless, important scientific questions remain to be addressed. For example, GQD-Nnps are ineffective at delivering puerarin. Future research should focus on enhancing the preparation technique to build more efficient GQD-Nnps-based drug delivery systems that fully realize their drug delivery potential. In addition, GQD-Nnps’ pharmacological properties, particularly their effects on UC and EIMs, require further in-depth investigation. Furthermore, it is critical to determine whether GQD-Nnps itself may be absorbed into the body and whether such absorption raises safety concerns.

## 4. Materials and Methods

### 4.1. Materials

Filters and dialysis bags were sourced from Shanghai Yuanye Biological Co., Ltd. (Shanghai, China). The bicinchoninic acid (BCA) protein assay kit was supplied by Shanghai Biyuntian Biotechnology Co., Ltd. (Shanghai, China). Ammonium formate and formic acid were purchased from Thermo Fisher Scientific (Waltham, MA, USA). Zoletil^®^50, a combination of tiletamine hydrochloride and zolazepam hydrochloride, was acquired from Virbac Trading (Shanghai) Co., Ltd. (Shanghai, China). Dimethyl sulfoxide (DMSO) was obtained from Merck (Kenilworth, NJ, USA), and methanol was bought from Honeywell Trading (Shanghai) Co., Ltd. (Shanghai, China). All solvents used were of HPLC grade or higher.

Reference compounds, including berberine, baicalein, baicalin, puerarin, glycyrrhizic acid, glycyrrhetinic acid, mycophenolic acid, and naringin, were purchased from Shanghai Yuanye Biological Co., Ltd. (Shanghai, China) or Dalian Meilun Biotechnology Co., Ltd. (Dalian, China), with purities exceeding 98%.

Dried pieces of *Pueraria lobata* (Willd.) Ohwi (batch 230718), *Scutellaria baicalensis* Georgi (batch 230807), *Coptis chinensis* Franch (batch 230517), and *Glycyrrhiza uralensis* Fisch. (batch 230614) were sourced from Shanghai Kangqiao Herbal Pieces Co., Ltd. (Shanghai, China) and authenticated according to the Pharmacopeia of the People’s Republic of China (2020 edition). The samples are deposited at Shanghai University of Traditional Chinese Medicine.

### 4.2. Preparation and Quality Control of GQD Water Extract

The GQD water extract was prepared in accordance with published procedures [20]. In summary, the herbs were subjected to two extractions with 10 volumes of boiling water. The dried herbal pieces of *Pueraria lobata* were initially decocted alone for 20 min, followed by a combined decoction with the herbal pieces of *Scutellaria baicalensis*, *Coptis chinensis*, and *Glycyrrhiza uralensis* at a weight ratio of 8:3:3:2 for an additional hour. The resulting extract was filtered through four layers of gauze, and the residue was re-extracted with 10 volumes of water for 1 h. The filtrates from both decoctions were pooled and vacuum-dried at 60 °C, yielding the GQD water extract powder.

For quality control of the GQD extract, a precise amount of GQD water extract powder was dissolved in a 50% (*v*/*v*) methanol aqueous solution to achieve a final concentration of 1 mg/mL. Quantification of 4 representative constituents (berberine, puerarin, baicalin, and glycyrrhizic acid) was performed using a validated and published liquid chromatography–tandem mass spectrometry (LC-MS/MS) method [20].

### 4.3. Preparation of GQD-Nnps

#### 4.3.1. Basic Protocol

GQD-Nnps powder was prepared through a series of procedures, including centrifugation, filtration, dialysis, and lyophilization. In short, the GQD water extract was dissolved in water to a concentration of 0.15 g/mL and sonicated for 1.5 h. The solution was then centrifuged at about 825 g for 10 min, and the supernatant was filtered through a 0.22-μm filter. The filtrate was dialyzed against a membrane with a 10.0 kDa cutoff for 3 days with stirring. The retentate was then lyophilized at −45 °C to yield GQD-Nnps powder.

#### 4.3.2. Optimization of the Isolation Protocol

In general, the concentration of GQD powder, dialysis time and stirring speed during dialysis have effects on the properties of the prepared GQD-Nnps. Therefore, a series of orthogonally designed experiments with three factors and three levels (Table 1) was carried out and replicated thrice to determine the optimum conditions for isolation.

### 4.4. Characterization of GQD-Nnps

#### 4.4.1. Dynamic Light Scattering (DLS) Analysis

The freeze-dried powder of GQD-Nnps was suspended in water at a concentration of 1 mg/mL and subjected to vigorous vortex mixing. Subsequently, the mixture was centrifuged at a speed of about 9200× *g* for a duration of 10 min. DLS measurements were then performed to ascertain the size and zeta potential of the nanoparticles present in the resulting supernatant, utilizing a Malvern Zetasizer Nano analyzer (Worcestershire, UK).

#### 4.4.2. Scanning Electron Microscopy (SEM) Analysis

Following gold spraying and vacuum drying, the morphological characteristics of the GQD-Nnps powder were examined utilizing an FEI Quanta 250 scanning electron microscope (Thermo Fisher Scientific, Hillsboro, OR, USA), which was operated at 10 kV.

#### 4.4.3. Contents of Protein and Polysaccharide

The GQD-Nnps powder was dispersed in an aqueous solution at a concentration of 1 mg/mL and subjected to sonication for 90 min. Subsequently, the protein content was quantified using the BCA assay, while the polysaccharide level was assessed via the phenol–sulfuric acid method.

For the BCA assay, the stock solution of BSA standard (2 mg/mL) was taken and serially two-fold diluted to obtain dilutions with concentrations of 1, 0.5, 0.25, 0.125, 0.063, and 0.031 mg/mL, respectively. A total of 100 μL of working solution (Solution A: Solution B = 50:1 mixed from the tBCA kit) was added to a 96-well plate, followed by the addition of 10 μL of BSA standard solution, pure water, or GQD-Nnps solution to each well. After thorough mixing, the mixture was incubated in a water bath at 37 °C for 30 min in the dark. The absorbance at 562 nm was then measured using a BioTek microplate reader (BioTek Instruments, Inc., Winooski, WV, USA). The protein concentration of GQD-Nnps solution was calculated based on the established standard curve of the BSA standard solution.

For the phenol–sulfuric acid method, 1 g of phenol was precisely weighed, and a 5% phenol solution was prepared by adding pure water. Exactly 5 mg of glucose powder was weighed, and a 1 mg/mL glucose standard stock solution was prepared by dissolving it in pure water and adjusting the volume to 5 mL. The glucose standard stock solution was diluted with pure water to prepare working solutions with concentrations of 0, 10, 20, 40, 100, and 200 μg/mL, respectively. A total of 200 µL of 5% phenol solution, 200 µL of pure water-diluted GQD-Nnps solution, or glucose standard working solution was precisely pipetted into 5 mL centrifuge tubes. To each tube, 1.5 mL of concentrated sulfuric acid was added, followed by thorough mixing. The mixture was transferred to a boiling water bath for 5 min of incubation. After cooling to room temperature, 200 µL of the mixture was transferred to a 96-well plate, and the absorbance at 485 nm was measured using the microplate reader. The total sugar concentration of the GQD-Nnps solution was calculated based on the standard curve plotted with the glucose standard solution.

#### 4.4.4. Contents and Encapsulation Efficiency of the GQD Constituents

Precisely measured lyophilized GQD-Nnps powder (denoted as M_total_) was reconstituted in water to achieve a final concentration of 1 mg/mL within a 4 mL volume. To ascertain the total amount of each constituent in the GQD-Nnps powder (designated as M_total constituent_), a 2 mL aliquot of the GQD-Nnps solution was combined with 8 mL of methanol and sonicated for 30 min. The resultant mixture was centrifuged at about 9200× *g* for 10 min, and the supernatant was subjected to LC-MS/MS analysis. M_total constituent_ was derived by multiplying the detected concentration by the solution volume. The content (W/W, %) of each constituent was calculated according to Equation (1).

Content = M_total constituent_/M_total_ × 100% (1)

The unbound amount of each constituent in the GQD-Nnps powder (referred to as M_free constituent_) was evaluated by transferring 0.5 mL of the GQD-Nnps solution into an ultrafiltration centrifuge tube featuring a 3 kDa molecular weight cutoff. Following centrifugation at about 7500× *g* for 20 min, the filtrate was collected and analyzed to determine the concentration of each constituent. M_free constituent_ was calculated based on the detected concentration and volume of the GQD-Nnps solution. The encapsulation efficiency of each constituent in GQD-Nnps was calculated according to Equation (2).

Encapsulation efficiency = (M_total constituent_ − M_free constituent_)/M_total constituent_ × 100% (2)

#### 4.4.5. In Vitro Stability of GQD-Nnps

The lyophilized powder of GQD-Nnps was precisely measured and resuspended to a 1 mg/mL concentration in either simulated gastric fluid (SGF; water with 2.0 g/L NaCl, 0.32% pepsin, pH 1.2) or simulated intestinal fluid (SIF; water with 0.2 mol/L sodium phosphate, 1% trypsin, pH 6.8) [43]. After centrifugation at about 9200× *g* for 10 min, the supernatant was retained for incubation at specified intervals (SGF: 0, 30, 60, 90, 120 min; SIF: 0, 1, 2, 4, 6 h). At each interval, aliquots of the supernatant were taken to promptly assess nanoparticle size and zeta potential.

#### 4.4.6. Fourier Transform Infrared Spectroscopy (FTIR) Analysis

A 10.0 mg sample of GQD-Nnps or an equivalent mixture of GQD constituents (berberine, baicalin, puerarin, and glycyrrhizic acid, proportioned according to their respective contents in GQD-Nnps) was intimately blended with 190 mg of potassium bromide (KBr) and subsequently pressed into discs. The FTIR spectra of the samples were then recorded using a Shimadzu IRAffinity-1 FTIR spectrometer (Kyoto, Japan) at a resolution of 4 cm^−1^ over the wavenumber range of 4000 to 500 cm^−1^.

#### 4.4.7. High-Resolution Mass Spectrometry (HRMS) Analysis

Approximately 5 mg of lyophilized GQD-Nnps powder was precisely weighed and dissolved in methanol–water (50:50, *v*/*v*) to a concentration of 2 mg/mL. The solution underwent ultrasonication for 30 min and was centrifuged at about 18,000× *g* for 10 min at 22 °C to obtain the supernatant.

HRMS analysis was performed using a Thermo Scientific Orbitrap Fusion Lumos Tribrid mass spectrometer with an ESI ion source. Chromatography was conducted on an ACQUITY UPLC BEH C_18_ column (100 × 2.1 mm, 1.7 µm). The mobile phase consisted of 0.05% formic acid in water (A) and 0.05% formic acid in acetonitrile (B). Gradient elution was as follows: 0–6 min, 10% B; 6–8 min, 50% B; 8–10 min, 80% B; 10–14 min, 95% B. The flow rate was 0.3 mL/min, column temperature was 40 °C, and injection volume was 5 µL. The autosampler was maintained at 4 °C. Positive ion mode was used with a scan range of *m*/*z* 50–1500. Capillary temperature was 350 °C, positive spray voltage 3.8 kV, sheath gas flow rate 45 arb, auxiliary gas flow rate 15 arb, and heated component temperature 300 °C. Secondary mass spectra were acquired in a data-dependent manner based on the precursor ion list.

### 4.5. Pharmacokinetics of GQD-Nnps in DSS-Induced UC Mice

#### 4.5.1. Mice

Male C57BL/6J mice (SPF, 22–24 g) were obtained from Beijing Vital River Laboratory Animal Technology Co. Ltd. (Beijing, China; SCXK (Beijing) 2021–0006). They were housed at the laboratory animal center of Shanghai University of Traditional Chinese Medicine (SHUTCM; SYXK (Shanghai) 2020–0009) under climate-controlled conditions (22–24 °C, 12 h light/dark cycle). Mice were fasted overnight with free access to water before experiments. All animal procedures were approved by the Institutional Animal Care and Use Committee of SHUTCM (number: PZSHUTCM211018015, date: 18 October 2021) and complied with its guidelines.

#### 4.5.2. Induction of UC in Mice

DSS was used to induce UC in mice according to the method described in the literature [44]. Briefly, 10 male C57BL/6J mice were assigned to the control group and provided with regular drinking water. Additionally, 135 male C57BL/6J mice were given a 3% DSS water solution, replaced every two days, for a continuous period of seven days. Mice’s body weight was monitored to assess the successful induction of UC.

#### 4.5.3. Drug Treatment

On day 8, UC mice were randomly assigned to three main groups according to body weight, with each group comprising nine subgroups (n = 5 per subgroup). The three main groups received intragastric administration of 6.1 g/kg GQD extract, a mixture of four constituents (equivalent to their respective doses in GQD extract, i.e., 0.247 g/kg puerarin, 0.108 g/kg berberine, 0.277 g/kg baicalin, and 0.028 g/kg glycyrrhizic acid), and 4.197 g/kg GQD-Nnps (containing 0.016 g/kg puerarin, 0.130 g/kg berberine, 0.235 g/kg baicalin, and 0.029 g/kg glycyrrhizic acid). At predetermined time points post-administration (0.083, 0.25, 0.5, 1, 2, 4, 8, 12, and 24 h), mice were anesthetized by intramuscular injection of Zoletil^®^ 50 (65 mg/kg), and blood was collected into heparin-coated tubes for plasma preparation; livers were excised, rinsed with distilled water, and flash-frozen in liquid nitrogen. The anesthetized mice were then sacrificed by cervical dislocation. Plasma and liver samples were stored at −80 °C. Liver homogenates were prepared with 5–10 times the volume of water. Concentrations of the four constituents and two metabolites (i.e., baicalein and glycyrrhetinic acid) in the biological samples were determined using the following LC-MS/MS method.

#### 4.5.4. Quantification of GQD Constituents in Biological Samples

Protein precipitation was achieved using three times the volume of acetonitrile, followed by quantification of the GQD constituents and metabolites in biological samples via our validated LC-MS/MS method [20]. The LC-MS/MS setup included an ACQUITY UPLC (Waters, Milford, MA, USA) with autosampler and an API QTRAP 6500+ mass spectrometer (Applied Biosystems, Foster City, CA, USA) with ESI source. Chromatography was conducted on an ACQUITY BEH C_18_ column (2.1 × 100 mm, 1.7 μm) from Waters (Shanghai, China). The mobile phase consisted of 5 mM ammonium acetate with 0.1% formic acid (Phase A) and methanol (Phase B). The gradient elution was programmed as: 0 min, 10% B; 1 min, 40% B; 5 min, 60% B; 10 min, 70% B; 12 min, 90% B; 18 min, 90% B; 18.1 min, 10% B; 20 min, 10% B. The flow rate was 0.3 mL/min, with column and autosampler temperatures at 40 °C and 4 °C, respectively. The injection volume was 2 μL. Mass spectrometry parameters were: ion-source temperature 500 °C; spray voltage 4500 V (+) or (−); atomization gas 50 psi; auxiliary gas 50 psi; curtain gas 35 psi. Naringin and mycophenolic acid served as positive and negative IS, respectively. Quantification was performed in multiple reaction monitoring (MRM) mode, with instrument parameters and linear ranges provided in Table S1.

#### 4.5.5. Pharmacokinetic Parameters Calculation

Non-compartmental model analysis was performed to obtain the pharmacokinetic parameters reflecting drug absorption and in vivo exposure levels, including the peak concentration (C_max_) and the area under the concentration–time curve (AUC_0–24 h_). Notably, the pharmacokinetic parameters of GQD constituents were calculated from mean concentrations across five mice at each time point.

### 4.6. Data Analysis

Except for the pharmacokinetic parameters, the results were expressed as mean ± standard deviation (SD), with statistical significance (*p* < 0.05 or 0.01) determined by one-way analysis of variance (ANOVA) followed by LSD or Dunnett’s test.

## 5. Conclusions

This study used an optimized strategy to isolate a unique Nnp from GQD extract, which was primarily composed of proteins and polysaccharides. The oral drug delivery system that GQD-Nnps create through complex interactions significantly enhances the colonic absorption and hepatic distribution of several small active constituents of GQD. In conclusion, the unique composition, formation mechanism, and pharmacokinetic characteristics of GQD-Nnps are highlighted in the work, indicating their significant potential for future research and development.

## Figures and Tables

**Table 1:** Results of the orthogonal experiment (mean ± SD, n = 3).

Tests	Factors			Size (nm)	Zeta Potential (mV)	Contents (mg/g)				Encapsulation Efficiency (%)			
	A	B	C			Ber	Bai	Pue	Gly	Ber	Bai	Pue	Gly
1	24	300	0.1	138.1 ± 9.2	−15.8 ± 1.7	23.8 ± 3.5	39.1 ± 4.0	0.9 ± 0.0	7.5 ± 0.3	97.8 ± 0.1	67.3 ± 4.6	4.7 ± 17.9	99.2 ± 0.6
2	24	400	0.15	103.9 ± 13.6	−14.7 ± 0.8	29.7 ± 2.1	57.3 ± 5.0	3.2 ± 0.5	7.3 ± 0.6	97.8 ± 0.2	69.0 ± 1.0	29.4 ± 4.3	99.2 ± 0.1
3	24	200	0.2	110.9 ± 8.1	−13.7 ± 1.5	30.9 ± 2.3	56.0 ± 3.0	3.8 ± 0.4	6.8 ± 1.0	99.1 ± 0.4	76.8 ± 3.1	40.5 ± 5.8	99.6 ± 0.1
4	48	300	0.15	119.9 ± 6.1	−17.6 ± 2.3	32.2 ± 0.8	35.4 ± 1.9	<0	4.7 ± 0.3	98.1 ± 0.1	81.4 ± 2.1	/	99.5 ± 0.1
5	48	400	0.2	114.1 ± 11.7	−17.6 ± 1.2	35.1 ± 5.0	37.6 ± 5.2	<0	4.9 ± 0.3	98.3 ± 0.3	82.7 ± 2.3	/	99.5 ± 0.2
6	48	200	0.1	118.0 ± 11.0	−16.2 ± 1.9	19.2 ± 1.8	19.9 ± 1.3	<0	5.3 ± 0.2	98.1 ± 0.3	73.3 ± 1.8	/	99.6 ± 0.1
7	72	300	0.2	101.4 ± 5.0	−18.7 ± 3.0	40.5 ± 4.2	41.5 ± 4.4	<0	4.7 ± 0.3	99.3 ± 0.0	92.0 ± 0.5	/	99.5 ± 0.1
8	72	400	0.1	134.1 ± 21.0	−19.8 ± 2.3	15.7 ± 2.8	15.5 ± 2.5	<0	3.9 ± 0.3	99.5 ± 0.5	87.4 ± 7.1	/	99.6 ± 0.0
9	72	200	0.15	118.7 ± 7.7	−16.5 ± 1.5	35.2 ± 6.8	35.2 ± 8.7	<0	3.6 ± 0.6	99.5 ± 0.1	94.0 ± 0.8	/	99.5 ± 0.0

A, dialysis time (h); B, stirring speed (rpm); C, concentration of GQD extract (g/mL). Ber, berberine; Bai, baicalin; Pue, puerarin; Gly, glycyrrhizic acid.

**Table 2:** Pharmacokinetic parameters of constituents in systemic circulation and livers of mice administered oral GQD extract, GQD-Nnps, or the GQD mixture (Mean, n = 5).

Treatments	Constituents	Circulation		Livers	
		C_max_ (h)	AUC_0–24 h_ (h·ng/mL)	C_max_ (h)	AUC_0–24 h_ (h·ng/mL)
GQD extract	Puerarin	2563.3	19,347.1	34,373.3	196,931.6
	Berberine	177.2	602.8	6396.0	36,406.2
	Baicalin	3182.5	20,048.3	ND	ND
	Baicalein	27.6	242.2	13,340.0	69,991.7
	Glycyrrhizic acid	69.2	627.8	ND	ND
	Glycyrrhetinic acid	175.0	1436.7	ND	ND
GQD-Nnps	Puerarin	502 (7751.1)	8386.4 (129,491.1)	17,961.6 (277,338.3)	75,783.1 (1,170,137.8)
	Berberine	121.2 (100.9)	1717.9 (1430.8)	8242.7 (6865.2)	49,209.3 (40,985.7)
	Baicalin	5686.7 (6706.0)	65,463.3 (77,197.4)	ND	ND
	Baicalein	38.0 (44.8)	504.6 (595.0)	25,520.0 (30,094.4)	122,109.7 (143,997.5)
	Glycyrrhizic acid	2196.3 (2088.2)	19,569.9 (18,606.3)	ND	ND
	Glycyrrhetinic acid	1018.7 (968.5)	9338.2 (8878.3)	ND	ND
GQD mixture	Puerarin	8173.5	79,511.8	12,322.7	94,513.8
	Berberine	107.0	439.3	6024.0	41,517.8
	Baicalin	2220.8	22,054.6	ND	ND
	Baicalein	19.6	239.1	3152.0	30,258.6
	Glycyrrhizic acid	4006.7	34,103.7	ND	ND
	Glycyrrhetinic acid	425.2	4556.3	ND	ND

ND, not detected. The data in parentheses were obtained by normalizing the experimental data to the dose of the corresponding constituents in the GQD extract.

## Data Availability

The original contributions presented in this study are included in the article/Supplementary Material. Further inquiries can be directed to the corresponding authors.

## Supplementary Materials

The following supporting information can be downloaded at: https://www.mdpi.com/article/10.3390/ph18111718/s1, Table S1. Instrument parameters of the liquid chromatography tandem mass spectrometry for 6 target analysts and the internal standards (IS).

## Abbreviations

5-ASAs, 5-aminosalicylates; AUC, area under the concentration–time curve; BCA, bicinchoninic acid; C_max_, peak concentration; C-T, concentration–time; DMSO, dimethyl sulfoxide; DAI, disease activity index; DLS, dynamic light scattering; DSS, dextran sulfate sodium; EIM, extraintestinal manifestation; FTIR, Fourier transform infrared spectroscopy; GQD, Gegen–Qinlian decoction; GQD-Nnps, natural nanoparticles in Gegen–Qinlian decoction; HRMS, high-resolution mass spectrometry; IBD, inflammatory bowel disease; LC-MS/MS, liquid chromatography–tandem mass spectrometry; Nnps, natural nanoparticles; SEM, scanning electron microscopy; SGF, simulated gastric fluid; SIF, simulated intestinal fluid; TCM, traditional Chinese medicine; UC, ulcerative colitis.
