# Correction: Mu et al. Natural Nanoparticles in Gegen–Qinlian Decoction Promote the Colonic Absorption of Active Constituents in Mice with Dextran Sulfate Sodium-Induced Ulcerative Colitis. *Pharmaceuticals* 2025, *18*, 1718

**DOI:** 10.3390/ph19040549

**Published:** 2026-03-30

**Authors:** Sheng Mu, Zhang-Jin Zheng, Jing-Ze Lu, Ling-Yun Pan, Bing-Liang Ma

**Affiliations:** 1 Department of Pharmacology, School of Pharmacy, Shanghai University of Traditional Chinese Medicine, Shanghai 201203, China; musheng0925@163.com (S.M.); zhangjinzheng2025@163.com (Z.-J.Z.); jingze_lu97@163.com (J.-Z.L.); 2 Experiment Center for Science and Technology, Shanghai University of Traditional Chinese Medicine, Shanghai 201203, China

## Error in Figure

In the original publication [1], there was a mistake in Figure 1B as published. The zeta potential histogram presented in Figure 1B was incorrectly displayed. The actual zeta potential values are negative (typically −13.7 ± 1.5 mV), but the histogram showed positive values. The corrected Figure 1 appears below.

The authors state that the scientific conclusions are unaffected. This correction was approved by the Academic Editor. The original publication has also been updated.


