# Supplementary material for: Natural Nanoparticles in Gegen–Qinlian Decoction Promote the Colonic Absorption of Active Constituents in Mice with Dextran Sulfate Sodium-Induced Ulcerative Colitis
Source: Pharmaceuticals (Basel). 2025 Nov 12;18(11):1718. doi: 10.3390/ph18111718 (PMC12654978; doi:10.3390/ph18111718)
Supplement: Supplementary file 1 [file pharmaceuticals-18-01718-s001.zip › pharmaceuticals-3950986-supplementary.pdf]

Supplementary materials

1 Supplementary tables

Table S1. Instrument parameters of the liquid chromatography tandem mass spectrometry for 6 target analysts and the internal standards (IS).

Constituents	m/z	Q1 (Da)	Q3 (Da)	DP (V)	CE (eV)	CXP (V)	Linear ranges (ng/mL)
Puerarin	[M+H]+	417.1	297.1	76.9	37.4	13.1	2.5-320.0
Baicalin	[M+H]+	447.4	271.1	46.9	26.0	17.8	5.0-640.0
Baicalein	[M+H]+	271.1	123.1	112.2	40.2	19.1	3.9-500.0
Berberine	[M]+	336.1	321.3	69.7	22.0	16.6	1.0-128.0
Glycyrrhizic acid	[M-H]-	821.4	351.1	-220.2	-55.4	-35.6	30.0-3840.0
Glycyrrhetinic acid	[M-H]-	469.3	355.0	-212.9	-63.4	-43.2	25.0-3200.0
Naringin (IS1)	[M+Na]+	603.2	457.1	130.8	33.9	21.1	/
Mycophenolic acid (IS2)	[M-H]-	319.0	191.0	-82.5	-30.2	-24.1	/

CE, collision energy; DP, declustering potential; CXP, collision cell exit potential.
